# Author Correction: A structure of the relict phycobilisome from a thylakoid-free cyanobacterium

**DOI:** 10.1038/s41467-023-44394-6

**Published:** 2023-12-19

**Authors:** Han-Wei Jiang, Hsiang-Yi Wu, Chun-Hsiung Wang, Cheng-Han Yang, Jui-Tse Ko, Han-Chen Ho, Ming-Daw Tsai, Donald A. Bryant, Fay-Wei Li, Meng-Chiao Ho, Ming-Yang Ho

**Affiliations:** 1 Department of Life Science, National Taiwan University, Taipei, Taiwan; 2 Institute of Biological Chemistry, Academia Sinica, Taipei, Taiwan; 3 Department of Anatomy, Tzu Chi University, Hualien, Taiwan; 4 Institute of Biochemical Sciences, National Taiwan University, Taipei, Taiwan; 5 Department of Biochemistry and Molecular Biology, The Pennsylvania State University, University Park, PA USA; 6 Boyce Thompson Institute, Ithaca, NY USA; 7 Plant Biology Section, Cornell University, Ithaca, NY USA; 8 Graduate Institute of Biochemistry and Molecular Biology, National Taiwan University, Taipei, Taiwan; 9 Institute of Plant Biology, National Taiwan University, Taipei, Taiwan

**Keywords:** Cryoelectron microscopy, Bacterial structural biology, Antenna complex, Bacterial evolution

Correction to: *Nature Communications* 10.1038/s41467-023-43646-9, published online 04 December 2023

The original version of this Article contained an error in Fig. 2a, in which an extraneous arrow was present. This has been corrected in both the PDF and HTML versions of the Article.

