# Supplementary material for: A structure of the relict phycobilisome from a thylakoid-free cyanobacterium
Source: Nat Commun. 2023 Dec 4;14:8009. doi: 10.1038/s41467-023-43646-9 (PMC10696076; doi:10.1038/s41467-023-43646-9)
Supplement: Supplementary file 1 — Supplementary Information [file 41467_2023_43646_MOESM1_ESM.pdf]

A structure of the relict phycobilisome from a thylakoid-free cyanobacterium

Han-Wei Jiang[1]†, Hsiang-Yi Wu[2]†, Chun-Hsiung Wang[2], Cheng-Han Yang[2], Jui-Tse Ko[1], Han-Chen Ho[3], Ming-Daw Tsai[2,4], Donald A. Bryant[5], Fay-Wei Li[6,7], Meng-Chiao Ho[2,4,8]*, Ming-Yang Ho[1,9]*

Affiliations:

[1]Department of Life Science, National Taiwan University; Taipei, Taiwan
[2]Institute of Biological Chemistry, Academia Sinica; Taipei, Taiwan
[3]Department of Anatomy, Tzu-Chi University; Hualien, Taiwan
[4]Institute of Biochemical Sciences, National Taiwan University; Taipei, Taiwan
[5]Department of Biochemistry and Molecular Biology, The Pennsylvania State University; University Park, PA, USA
[6]Boyce Thompson Institute; Ithaca, NY, USA
[7]Plant Biology Section, Cornell University; Ithaca, NY, USA
[8]Graduate Institute of Biochemistry and Molecular Biology, National Taiwan University Taipei, Taiwan.
[9]Institute of Plant Biology, National Taiwan University; Taipei, Taiwan

*Corresponding authors. Email: mingyang@ntu.edu.tw; joeho@gate.sinica.edu.tw

†These authors contributed equally to this work

The PDF file includes:

Supplementary Figs. 1 to 16
Supplementary Tables 1 to 6

Supplementary Figures

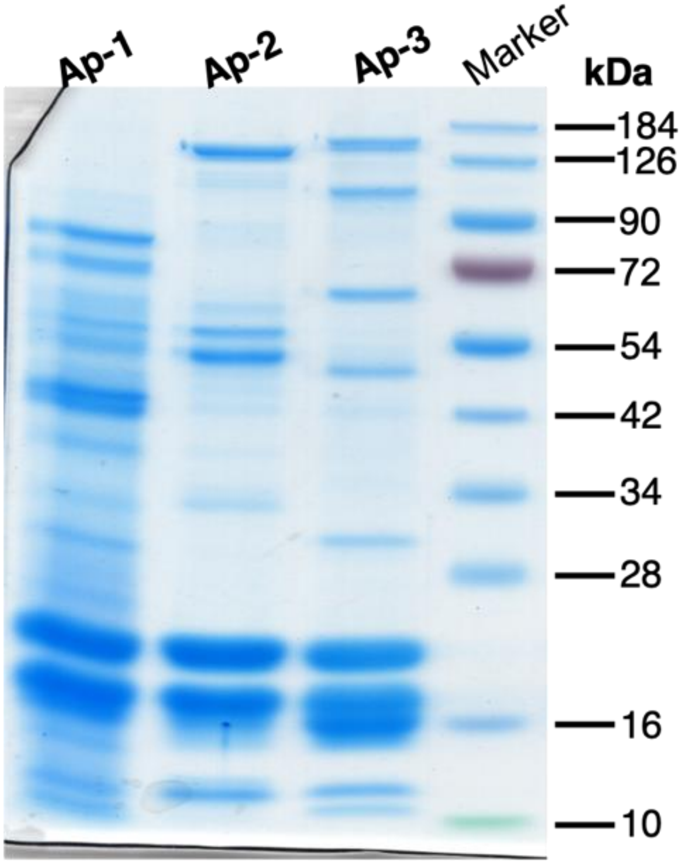

Supplementary Fig. 1
SDS-PAGE of isolated phycobiliproteins and phycobilisome fractions described in Fig. 1. The gel was stained with Coomassie Blue G-250. Protein mass standards (kDa) were labeled on the left. The concentration of acrylamide was 8-20 % (w/v). Experiments were repeated more than three times with similar results. Source data are provided as a Source Data file.

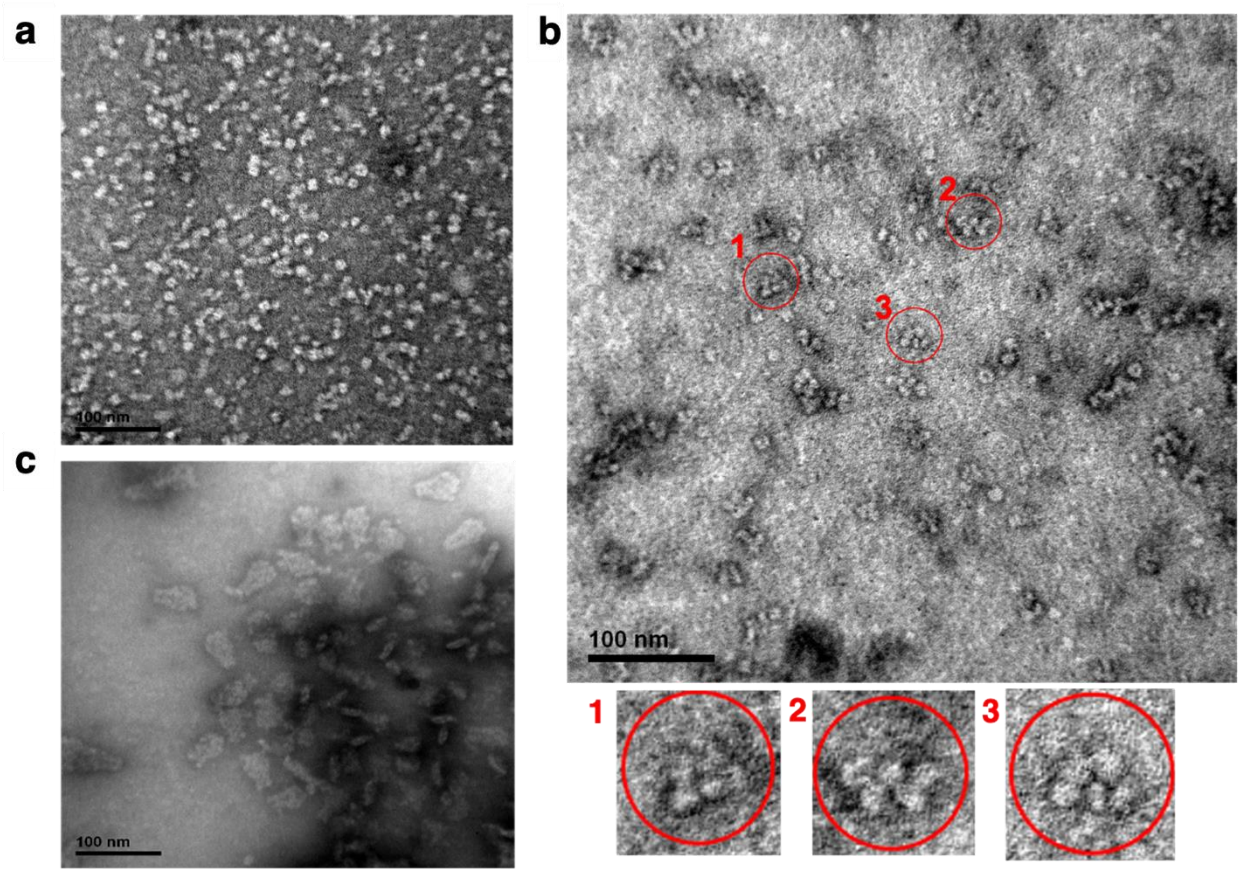

Supplementary Fig. 2

Negative staining EM of PBS fractions of *A. panamensis*.

a Negative staining EM of the Ap-1 fraction from Fig.1b. Dissociated PBS was found in the Ap-1 fraction. **b** Negative staining EM of the Ap-2 fraction from Fig.1b. The particles showing pentacylindrical cores are indicated by red circles and enlarged in the bottom panels. **c** Negative staining EM of the Ap-3 fraction from Fig.1b. Experiments were repeated more than three times with similar results.

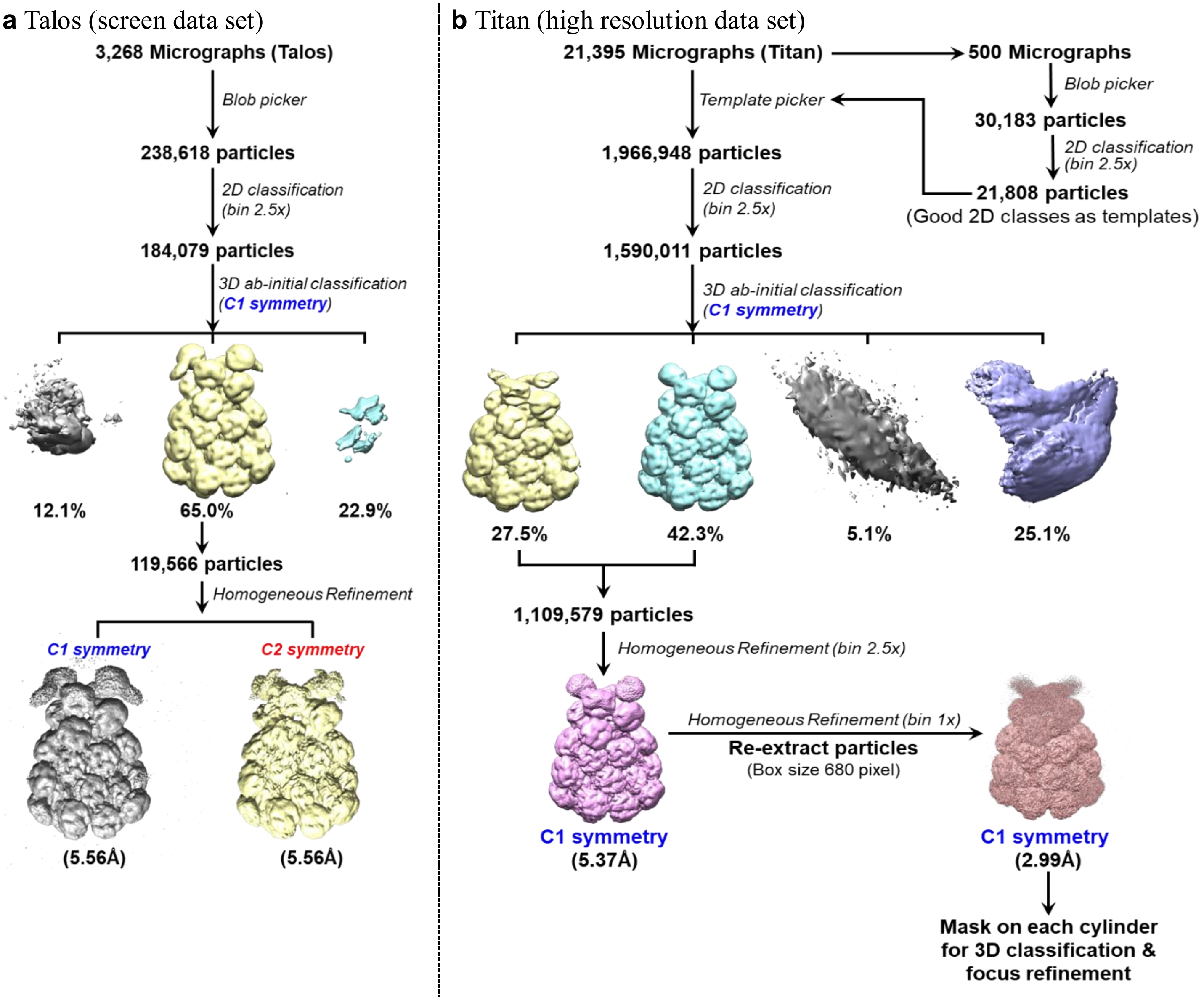

a Talos (screen data set)
3,268 Micrographs (Talos)
Blob picker
238,618 particles
2D classification
(bin 2.5x)
184,079 particles
3D ab-initial classification
(C1 symmetry)
12.1%
65.0%
22.9%
119,566 particles
Homogeneous Refinement
C1 symmetry
C2 symmetry
(5.56Å)
(5.56Å)
b Titan (high resolution data set)
21,395 Micrographs (Titan)
500 Micrographs
Blob picker
30,183 particles
2D classification
(bin 2.5x)
21,808 particles
(Good 2D classes as templates)
Template picker
1,966,948 particles
2D classification
(bin 2.5x)
1,590,011 particles
3D ab-initial classification
(C1 symmetry)
27.5%
42.3%
5.1%
25.1%
1,109,579 particles
Homogeneous Refinement (bin 2.5x)
Homogeneous Refinement (bin 1x)
Re-extract particles
(Box size 680 pixel)
C1 symmetry
(5.37Å)
C1 symmetry
(2.99Å)
Mask on each cylinder
for 3D classification &
focus refinement

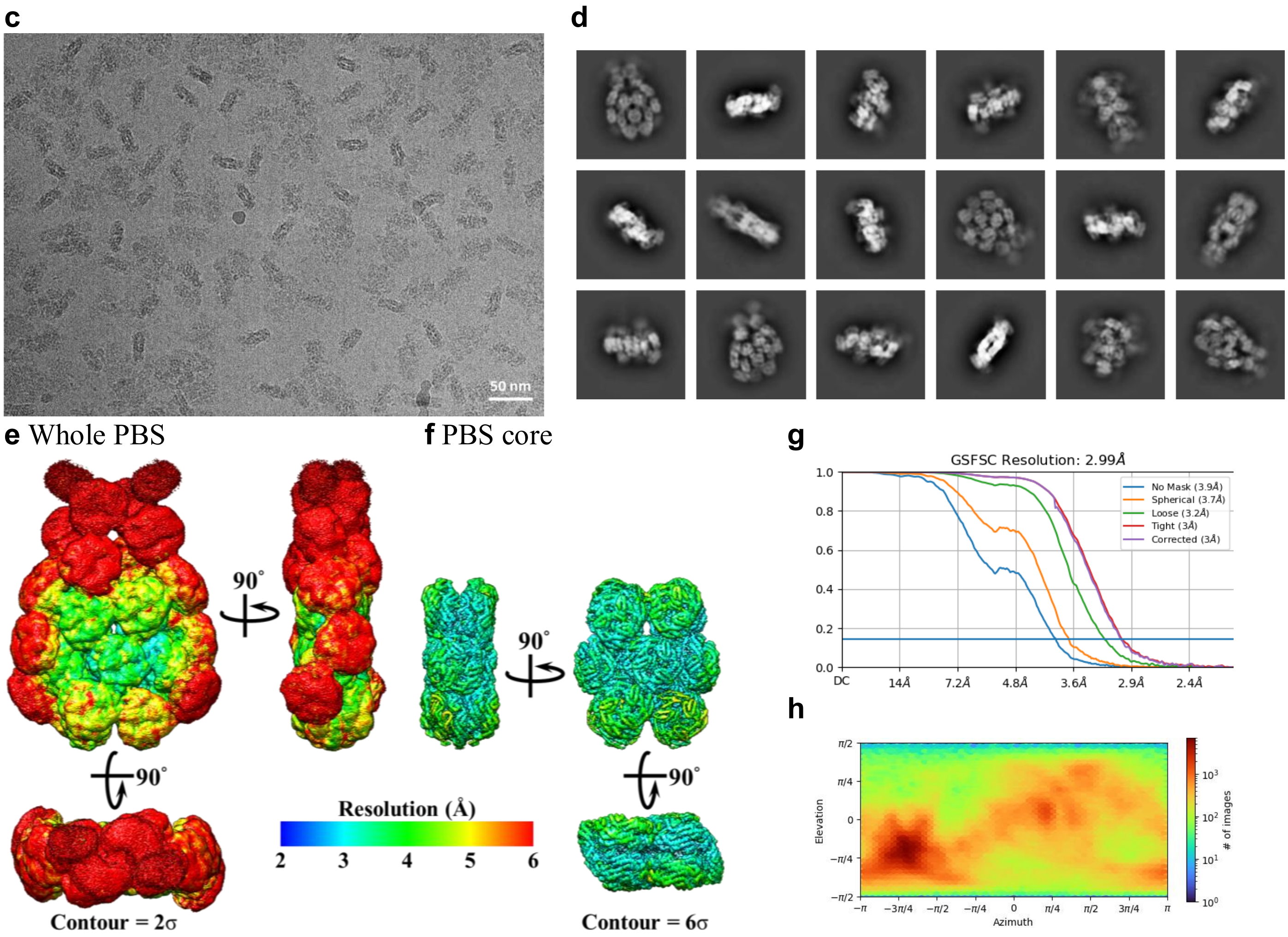

Supplementary Fig. 3

Cryo-EM data processing workflow and 3D reconstruction. a The data processing workflow of Talos data set. **b** The data processing workflow of high resolution 300 kV Titan data set. **c** A typical motion-corrected electron micrograph of PBS. The scale bar corresponds to 50 nm. Experiments were repeated more than three times with similar results. **d** 2D classification of the data from cryo-EM results in high-resolution classes showing different views of the PBS. **e** Resolution maps for the final reconstructions of whole PBS and **f** PBS core (colored according to the local resolution). **g** Gold standard FSC plots for the 3D reconstructions. **h** Euler angle distribution of the particle images.

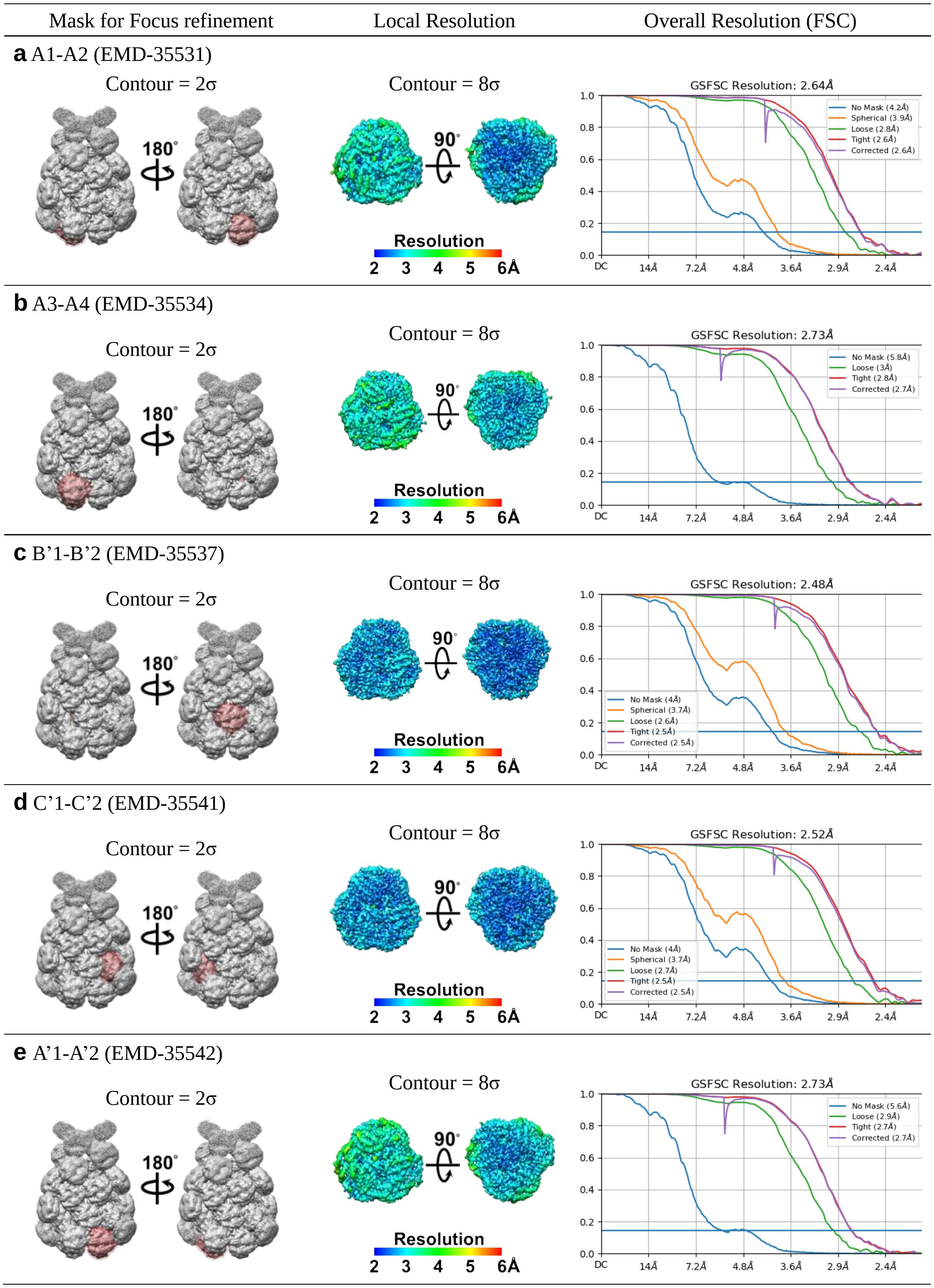
Mask for Focus refinement
Local Resolution
Overall Resolution (FSC)
a A1-A2 (EMD-35531)
Contour = 2σ
180°
Contour = 8σ
90°
Resolution
2 3 4 5 6Å
GSFSC Resolution: 2.64Å
No Mask (4.2Å)
Spherical (3.9Å)
Loose (2.8Å)
Tight (2.6Å)
Corrected (2.6Å)
b A3-A4 (EMD-35534)
Contour = 2σ
180°
Contour = 8σ
90°
Resolution
2 3 4 5 6Å
GSFSC Resolution: 2.73Å
No Mask (5.8Å)
Loose (3Å)
Tight (2.8Å)
Corrected (2.7Å)
c B'1-B'2 (EMD-35537)
Contour = 2σ
180°
Contour = 8σ
90°
Resolution
2 3 4 5 6Å
GSFSC Resolution: 2.48Å
No Mask (4Å)
Spherical (3.7Å)
Loose (2.6Å)
Tight (2.5Å)
Corrected (2.5Å)
d C'1-C'2 (EMD-35541)
Contour = 2σ
180°
Contour = 8σ
90°
Resolution
2 3 4 5 6Å
GSFSC Resolution: 2.52Å
No Mask (4Å)
Spherical (3.7Å)
Loose (2.7Å)
Tight (2.5Å)
Corrected (2.5Å)
e A'1-A'2 (EMD-35542)
Contour = 2σ
180°
Contour = 8σ
90°
Resolution
2 3 4 5 6Å
GSFSC Resolution: 2.73Å
No Mask (5.6Å)
Loose (2.9Å)
Tight (2.7Å)
Corrected (2.7Å)
1.0
0.8
0.6
0.4
0.2
0.0
DC
14Å
7.2Å
4.8Å
3.6Å
2.9Å
2.4Å

Mask for Focus refinement	Local Resolution	Overall Resolution (FSC)

f A’3-A’4 (EMD-35544)

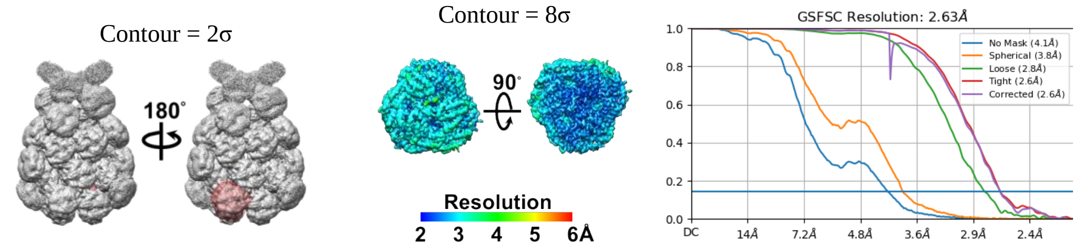

g B1-B2 (EMD-35546)

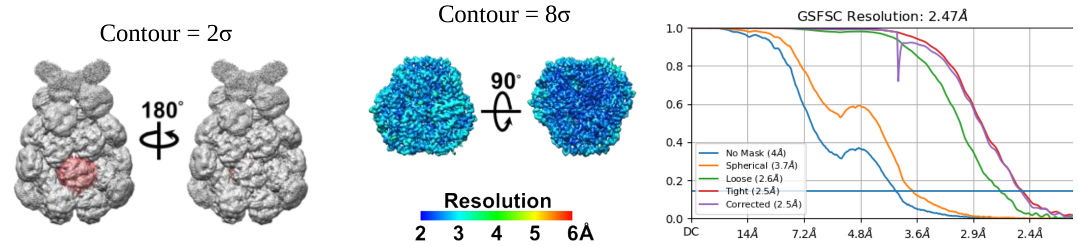

h C1-C2 (EMD-35548)

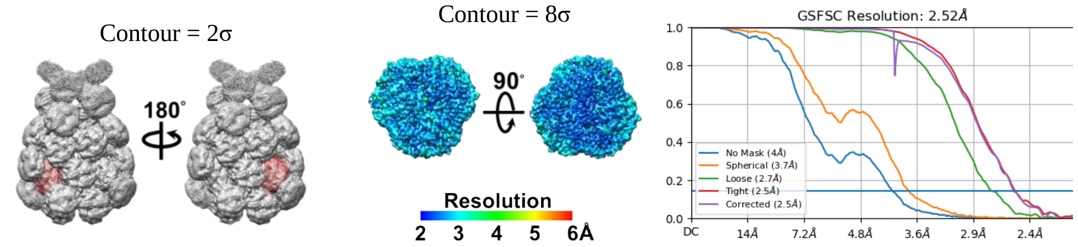

i D3-D4 (EMD-35549)

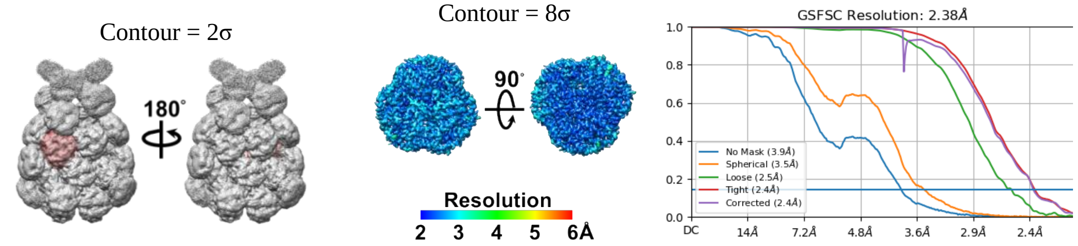

j D1-D2 (EMD-35550)

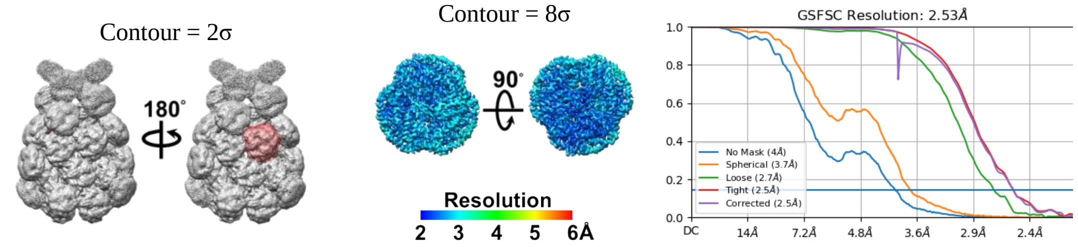

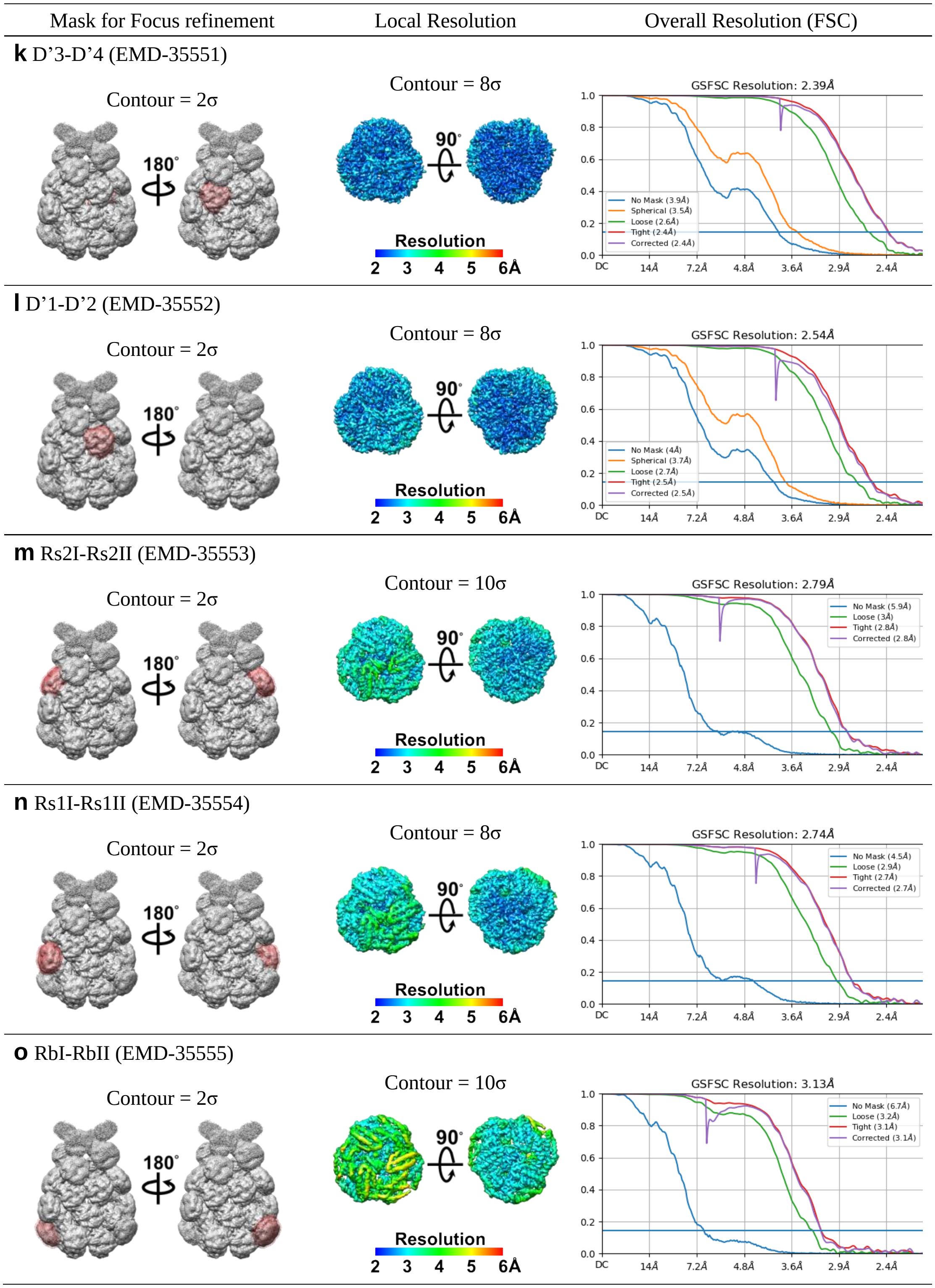

Mask for Focus refinement
Local Resolution
Overall Resolution (FSC)
k D'3-D'4 (EMD-35551)
Contour = 2σ
180°
Contour = 8σ
90°
Resolution
2 3 4 5 6Å
GSFSC Resolution: 2.39Å
No Mask (3.9Å)
Spherical (3.5Å)
Loose (2.6Å)
Tight (2.4Å)
Corrected (2.4Å)
l D'1-D'2 (EMD-35552)
Contour = 2σ
180°
Contour = 8σ
90°
Resolution
2 3 4 5 6Å
GSFSC Resolution: 2.54Å
No Mask (4Å)
Spherical (3.7Å)
Loose (2.7Å)
Tight (2.5Å)
Corrected (2.5Å)
m Rs2I-Rs2II (EMD-35553)
Contour = 2σ
180°
Contour = 10σ
90°
Resolution
2 3 4 5 6Å
GSFSC Resolution: 2.79Å
No Mask (5.9Å)
Loose (3Å)
Tight (2.8Å)
Corrected (2.8Å)
n Rs1I-Rs1II (EMD-35554)
Contour = 2σ
180°
Contour = 8σ
90°
Resolution
2 3 4 5 6Å
GSFSC Resolution: 2.74Å
No Mask (4.5Å)
Loose (2.9Å)
Tight (2.7Å)
Corrected (2.7Å)
o RbI-RbII (EMD-35555)
Contour = 2σ
180°
Contour = 10σ
90°
Resolution
2 3 4 5 6Å
GSFSC Resolution: 3.13Å
No Mask (6.7Å)
Loose (3.2Å)
Tight (3.1Å)
Corrected (3.1Å)

Mask for Focus refinement	Local Resolution	Overall Resolution (FSC)

p Rs2’ I-Rs2’II (EMD-35556)

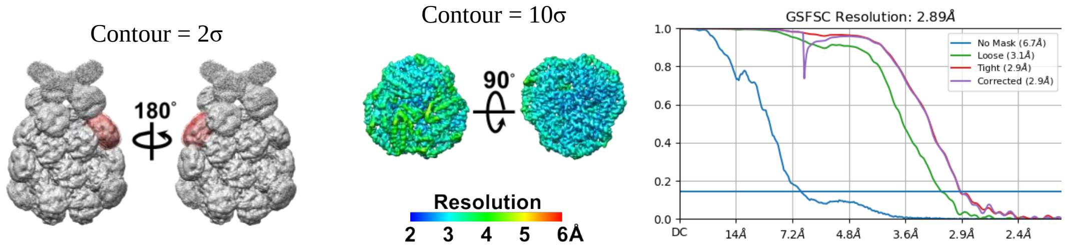

q Rs1’I-Rs1’II (EMD-35557)

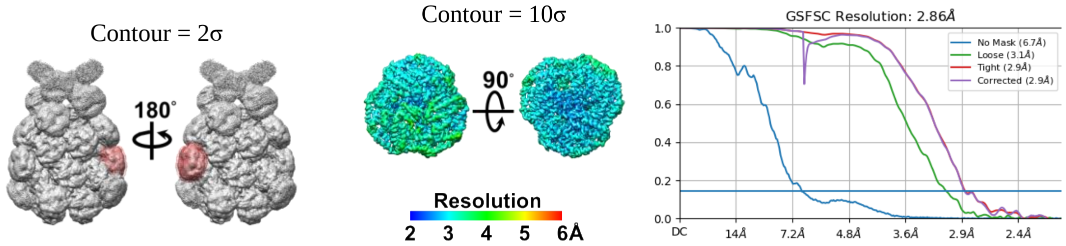

r Rb’I-Rb’II (EMD-35558)

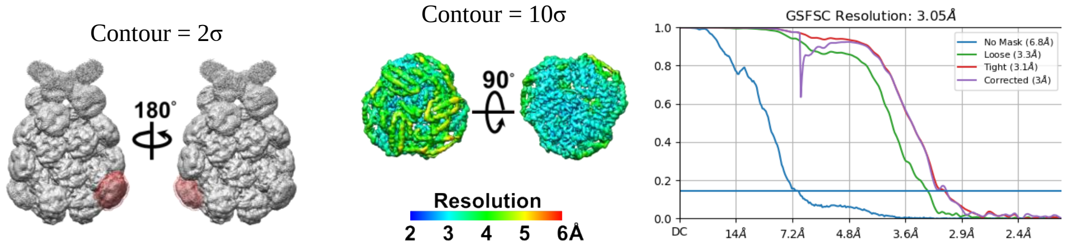

s Rt1I-Rt1II (EMD-35559)

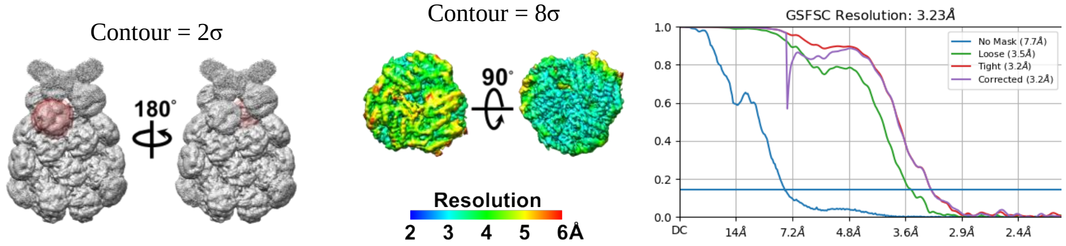

t Rt2I-Rt2II (EMD-35560)

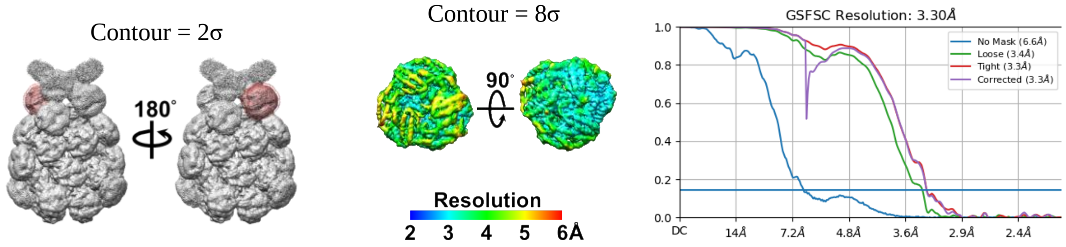

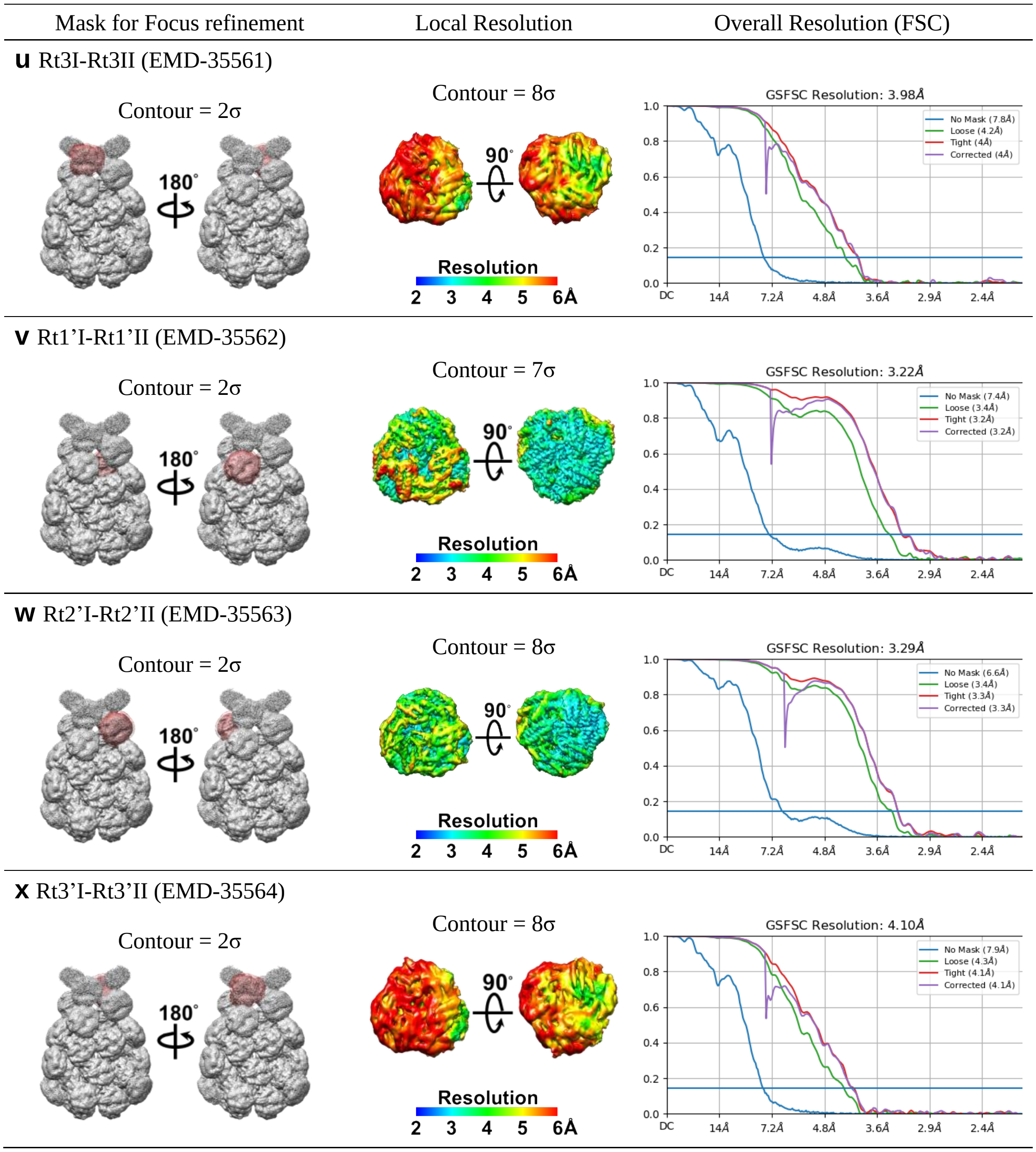

Supplementary Fig. 4

Overall and local resolution estimation for each 3D focus refinement. a to **x** show the area of focus refinement. **Left panel:** The masks used for the 3D focus refinement. The cryo-EM density maps were shown as gray, and the mask used for focus refinement was shown as transparent red. **Middle panel:** Resolution maps for the 3D focus-refined reconstructions. The densities were colored according to the local resolution calculated by cryoSPARC. **Right panel:** Gold-standard FSC plots for the 3D focus-refined reconstructions. The global resolutions were estimated by gold-standard FSC (threshold at 0.143).

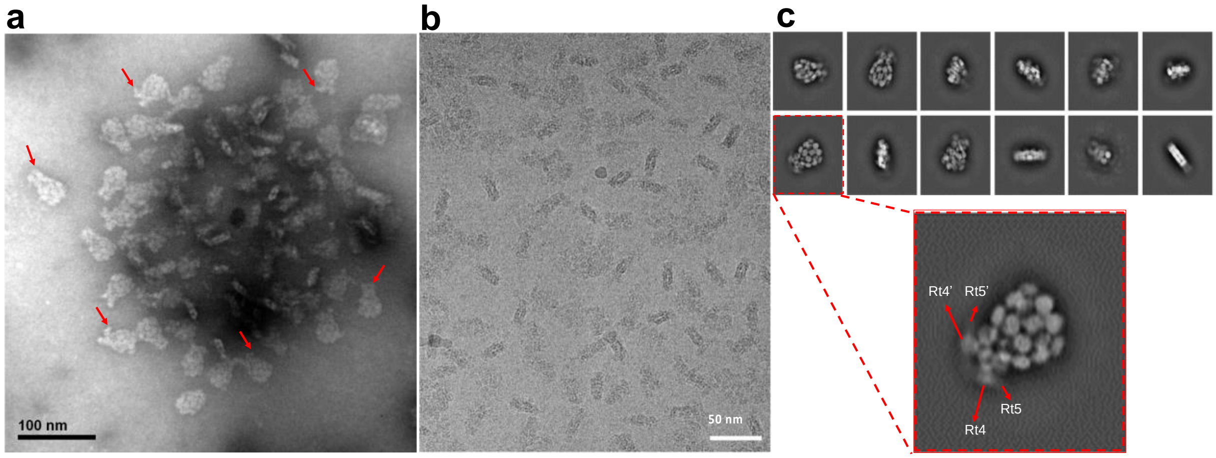

Supplementary Fig. 5

Negative-staining EM and two-dimensional (2D) averages from cryo-EM images of PBS from *A. panamensis.*

a A micrograph of negatively stained PBS complexes flexible rod components Rt4/Rt4’ and Rt5/Rt5’ are denoted by arrows. Experiments were repeated more than three times with similar results. The scale bar corresponds to 100 nm. **b** A typical motion-corrected electron micrograph of PBS. The scale bar corresponds to 50 nm. **c** 2D classification of the data from cryo-EM results in high-resolution classes showing different views of the PBS. The zoom-in view shows the flexible PC hexamers Rt4/Rt4’ and Rt5/Rt5’ in the chains (denoted by arrows).

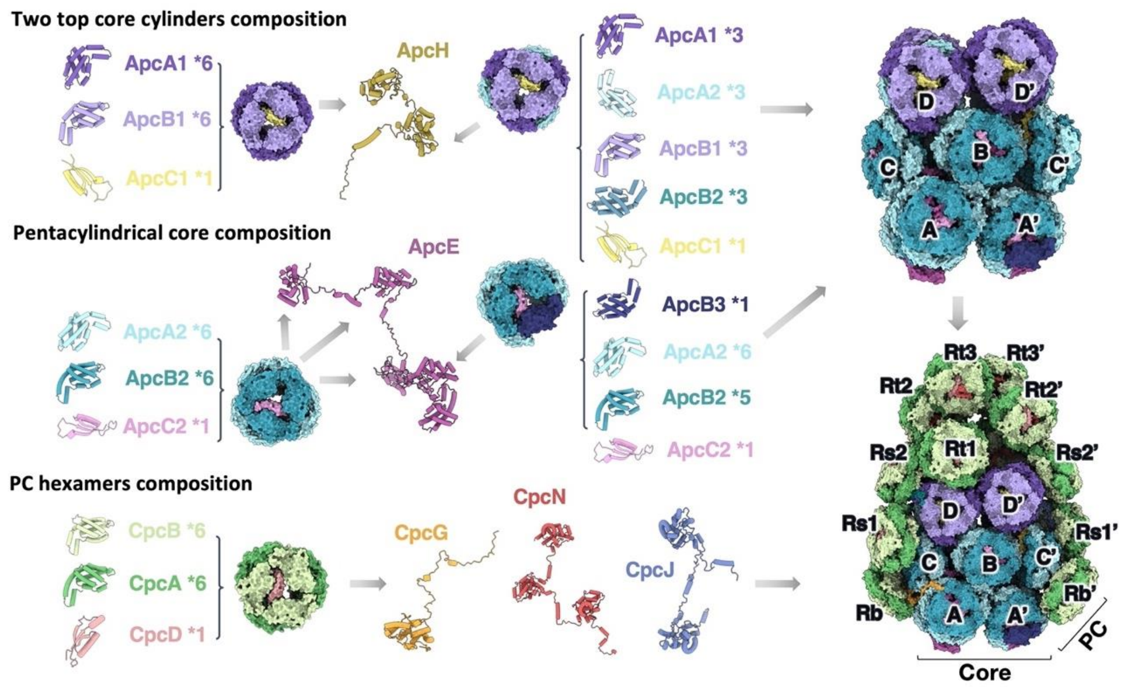

Supplementary Fig. 6

Schematic model of the PBS assembly and architecture.

Besides the C/C’ cylinders, the core cylinders comprise four stacked trimers; each trimer contains three α- and three β-AP subunits that form two $(\alpha\beta)_6$ hexamers. The two top core cylinders are assembled with the ApcA1, ApcB1, and ApcC1 and connected to the pentacylindrical core by ApcH. The pentacylindrical core is assembled with ApcA2, ApcB2, and ApcC2 and connected by ApcE. Only the A/A’ cylinders have one copy of β subunit replaced by ApcB3. In the basal cylinders of the core, ApcE protrudes toward the membrane and is likely critical to the interaction between PBS and PSII. Each PC hexamer is assembled with CpcA, CpcB, and CpcD and connected to the core by three linkers (CpcG, CpcJ, and CpcN).

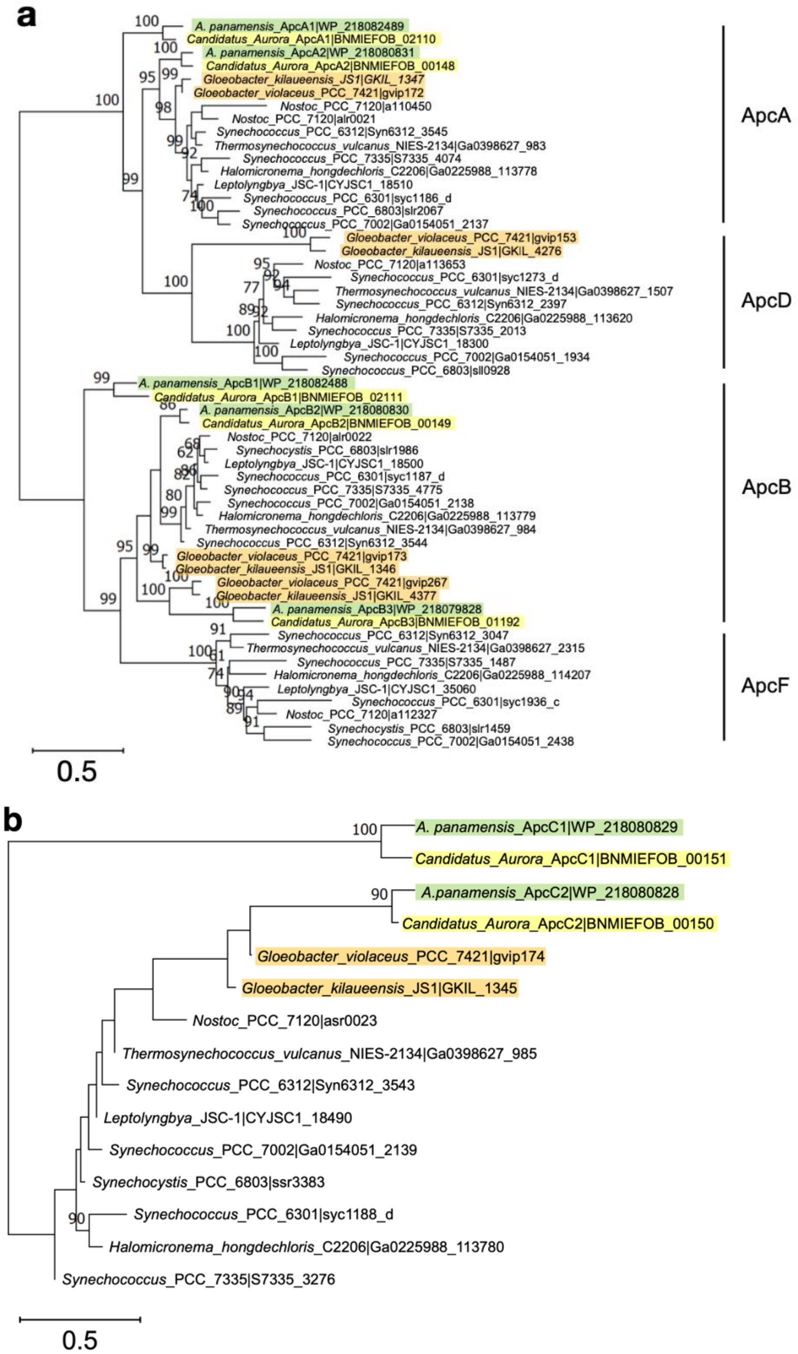

Supplementary Fig. 7

Maximum likelihood (ML) phylogenies of ApcA, ApcB, ApcC, ApcD, and ApcF.

The phylogenetic tree is constructed using the full length of ApcA, ApcB, ApcD, and ApcF (**a**) and ApcC (**b**) protein sequences from *Gloeobacter* spp., Aurora, *A. panamensis,* and crown cyanobacteria with the best-fitting amino acid substitution model LG + F + G4 selected by ModelFinder[59]. Bootstrap values are presented on the tree nodes, while only values larger than 50 are shown. The scale bars indicate the number of substitutions per site. Proteins in *Gloeobacter* spp., Aurora, and *A. panamensis* are highlighted in orange, yellow, and green, respectively. Unhighlighted proteins belong to crown Cyanobacteria.

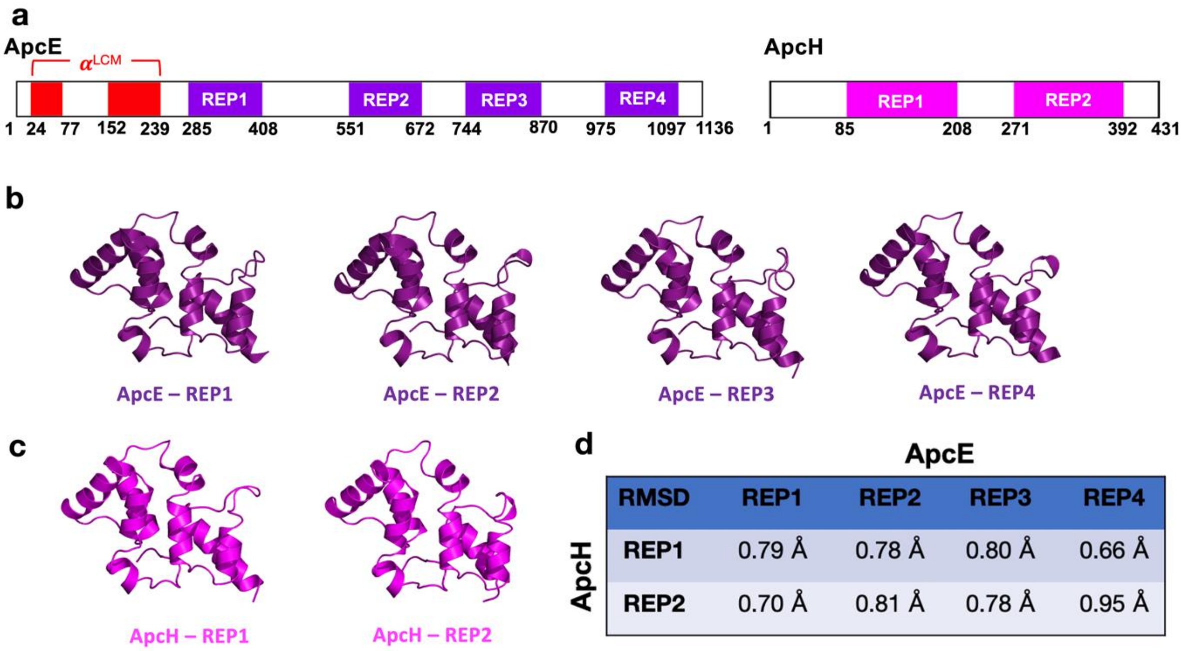

RMSD	REP1	REP2	REP3	REP4
REP1	0.79 Å	0.78 Å	0.80 Å	0.66 Å
REP2	0.70 Å	0.81 Å	0.78 Å	0.95 Å

Supplementary Fig. 8

Structural comparison of REP (pfam00427) domains in ApcE and ApcH in *A. panamensis*. **a**, Diagram of the positions of REP domains in ApcE and ApcH. **b**, Cartoon representation of the REP1 to REP4 domains in ApcE (purple) and **c**, the REP1 and REP2 domains in ApcH (magenta). **d**, The calculated RMSD values between the REP domains in ApcE and ApcH. Overall, all these REP domains are structurally similar.

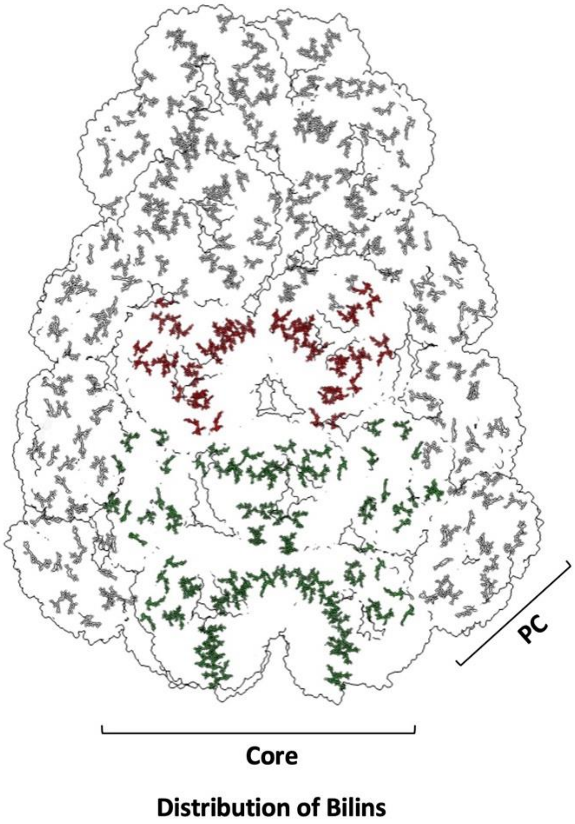

Supplementary Fig. 9

Bilin distribution in the PBS from *A. panamensis*. All bilins are shown in stick representation and color-coded according to their spatial locations. Bilins in PC hexamers, the top two core cylinders, and the pentacylindrical core are colored grey, red, and green, respectively.

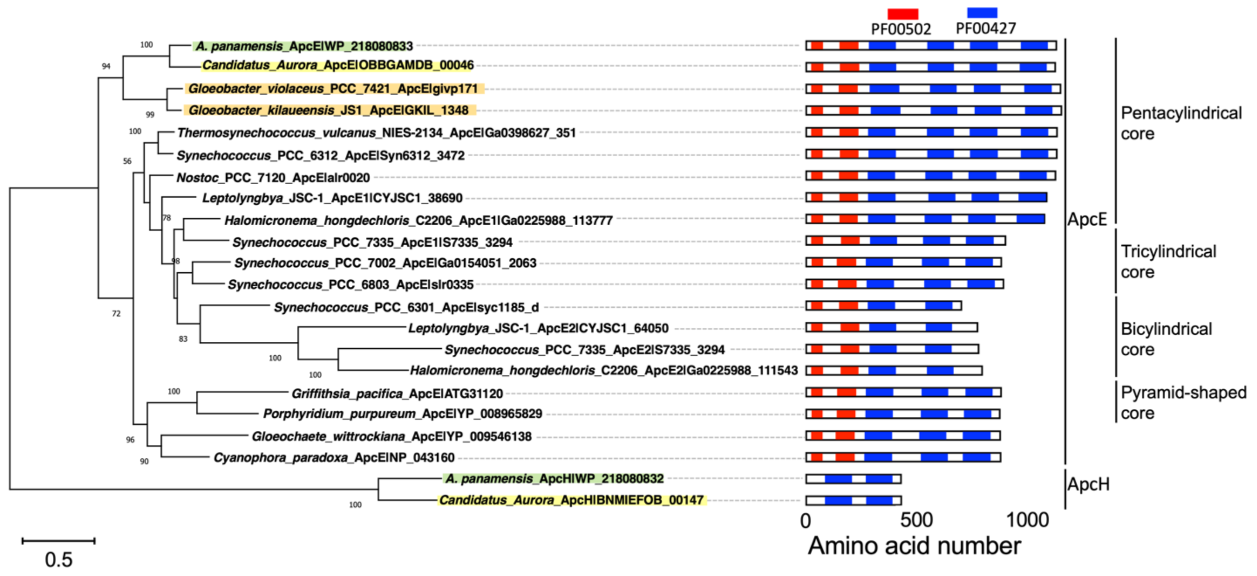

Supplementary Fig. 10

Core linker protein phylogeny. The maximum likelihood phylogenetic tree reconstructed using the full-length ApcE or ApcH protein sequences from *Gloeobacter* spp. (orange), Aurora (yellow), *A. panamensis* (green), glaucophyta, rhodophyta[5], and crown Cyanobacteria. Bootstrap values are presented on the tree nodes; only the values higher than 50 are shown. The scale bars indicate the number of substitutions per site.

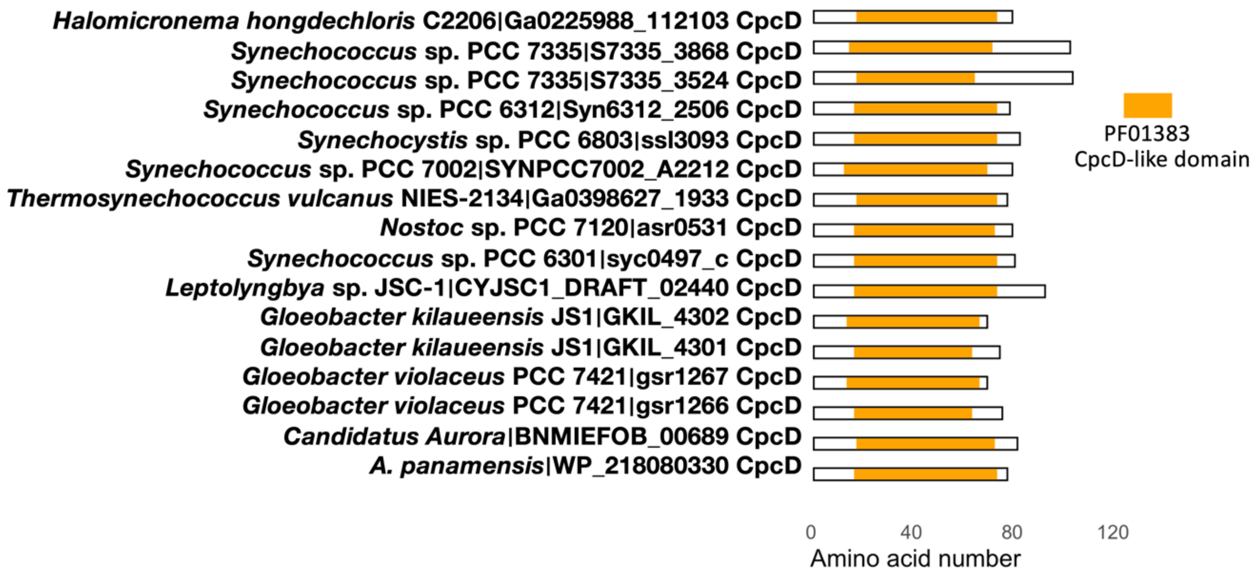

Supplementary Fig. 11

Schematic domain architecture of CpcD proteins in *A. panamensis*, Aurora, *Gloeobacter* spp., and crown cyanobacteria. The domain architecture of the proteins was identified by using the GenomeNet Bioinformatics tool-MOTIF (https://www.genome.jp/tools/motif/).

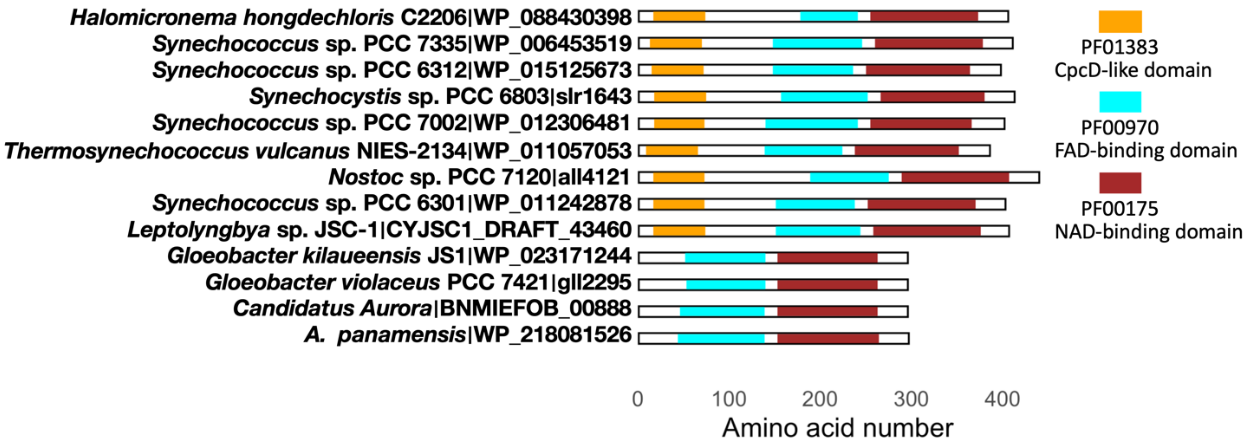

Supplementary Fig. 12

Schematic domain architecture of FNR proteins in *A. panamensis,* Aurora, *Gloeobacter* spp., and crown cyanobacteria. The domain architecture of the proteins was identified by using the GenomeNet Bioinformatics tool-MOTIF (https://www.genome.jp/tools/motif/).

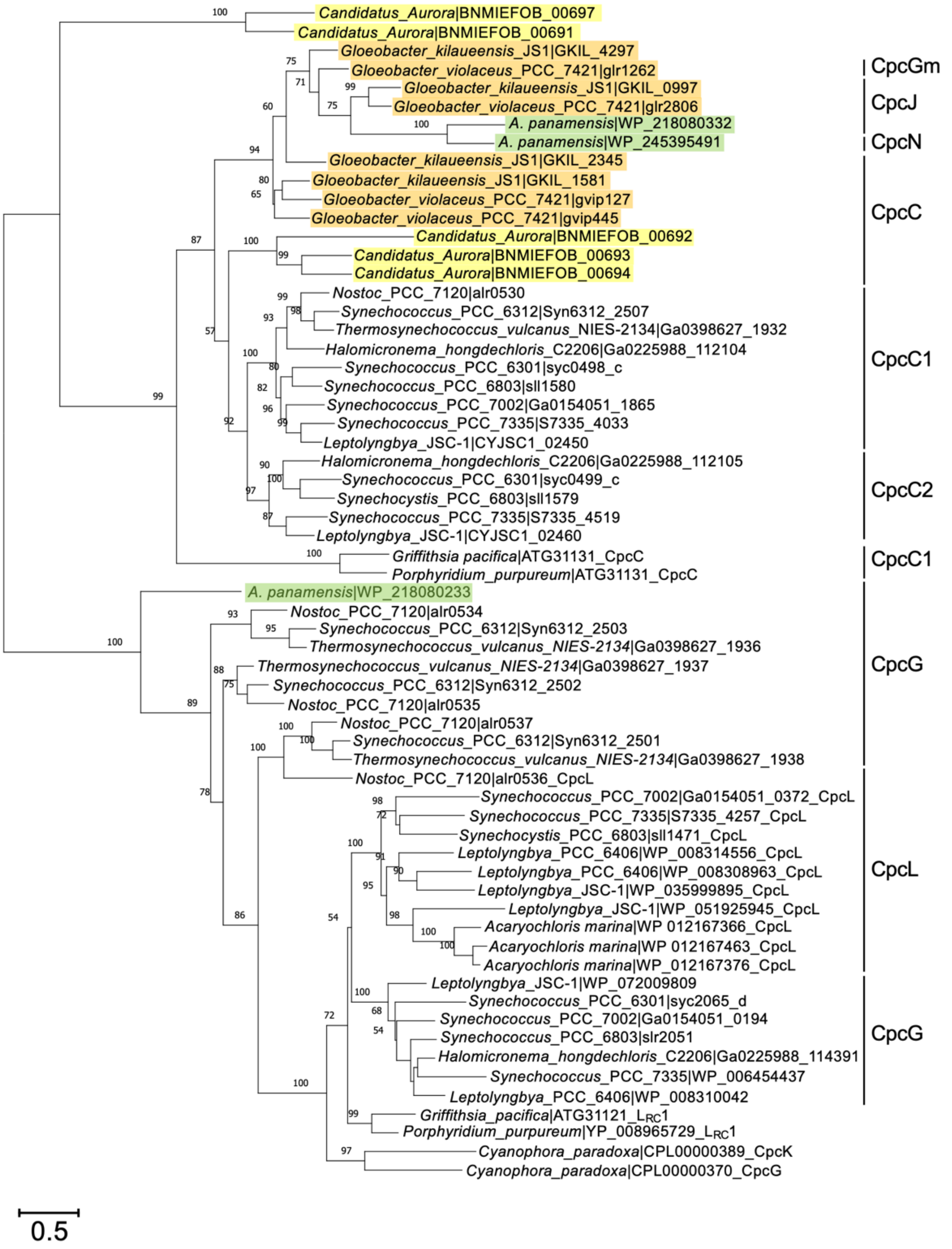

Supplementary Fig. 13

Phylogenetic relationship of the PC hexamer linker proteins

The maximum likelihood phylogenetic tree reconstructed using the full-length protein sequences of PC hexamer linker proteins from *Gloeobacter* spp. (orange), Aurora (yellow), *A. panamensis* (green), *Cyanophora paradoxa*[65], rhodophyte, and crown Cyanobacteria. Bootstrap values are presented on the tree nodes; only the values higher than 50 are shown. The scale bars indicate the number of substitutions per site.

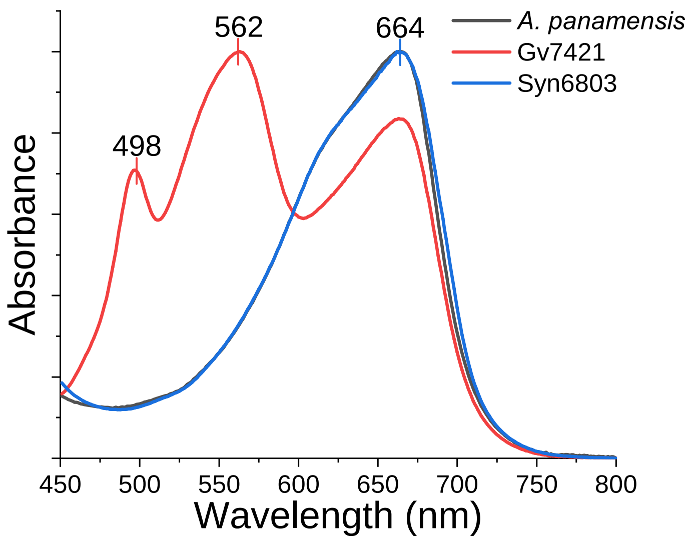

Supplementary Fig. 14

Phycobiliproteins from *A. panamensis* only carry phycocyanobilin (PCB) chromophores. Absorption spectra of PBS samples isolated from *A. panamensis* (black line), Gv7421 (red line), and Syn6803 (blue line) were taken in 8.0 M urea at pH 3.0 to denature PBSs and protonate chromophores[66]. The 562 nm and 498 nm peaks indicate phycoerythrobilin and phycourobilin, respectively[20]. Phycocyanobilin has an absorbance maximum at 664 nm under these conditions[56]. Source data are provided as a Source Data file.

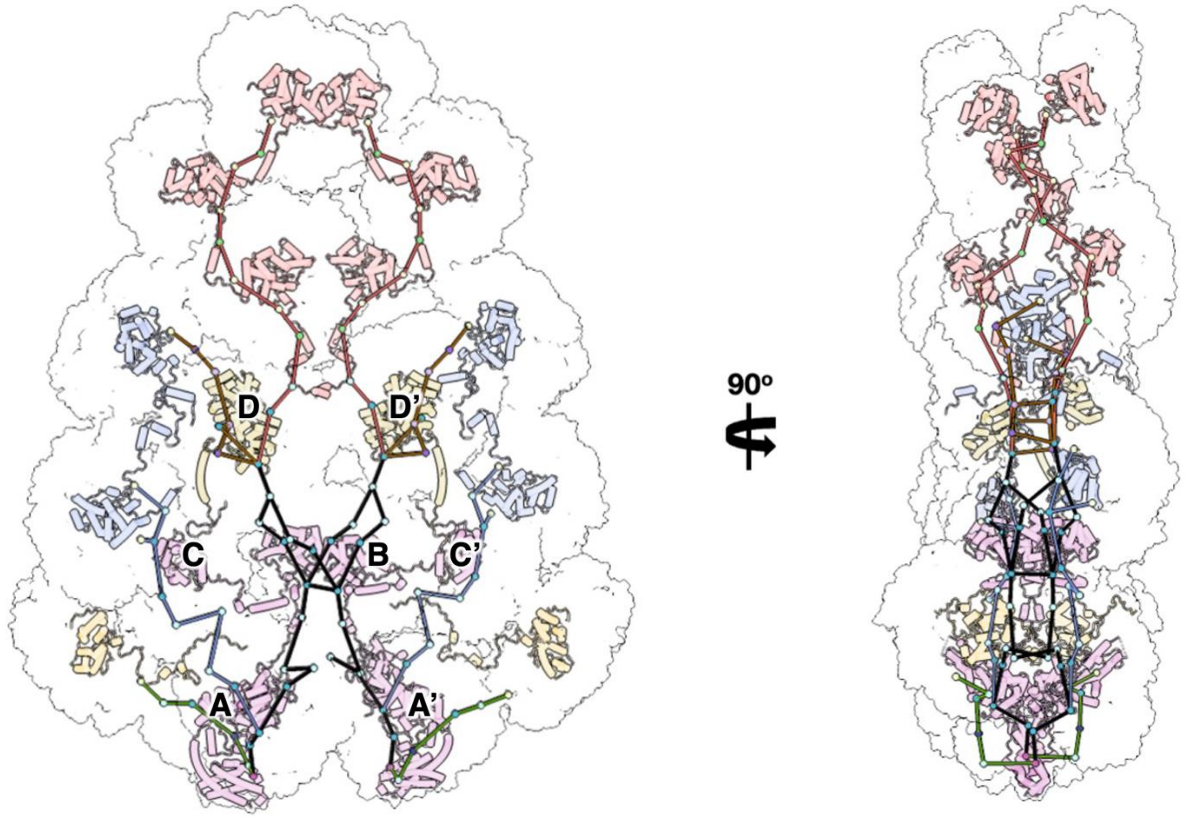

Supplementary Fig. 15
Front and side views of plausible excitation energy transfer (EET) pathways of the paddle-shaped PBS.
This figure depicts front (left panel) and side (right panel) views of Fig. 6a. Key bilins and corresponding linker proteins in the EET pathways based on the shortest bilin distances are shown as dots and cartoon diagrams, respectively. Each bilin's 10th C atom (the central carbon between the rings B and C of the bilin) is shown as a dot. Dots are colored according to their protein subunits, as shown in Fig. 2h, and bilin distances were calculated based on the positions of dots.

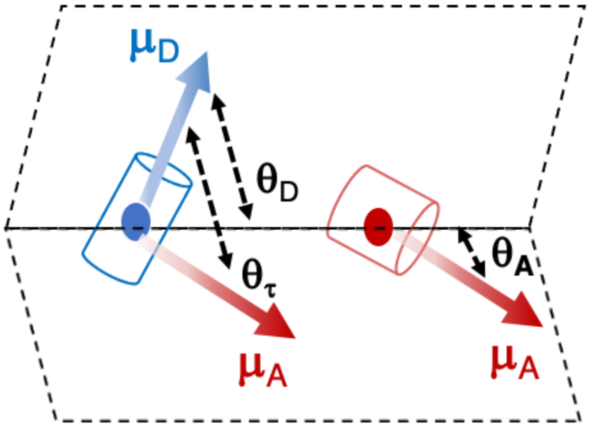

Supplementary Fig. 16

The factors used to estimate the orientation factors between chromophores.

The transition dipole moment of the donor and acceptor are μ_D (blue arrow) and μ_A (red arrow), respectively. θ_D and θ_A are the angles between D–A connecting line, and μ_D and μ_A. θ_τ is the angle between μ_D and μ_A. This figure has been drawn by using Supplementary Fig. 11 in Kawakami, K. et al., 2022[17] as a reference.

Supplementary Tables

Supplementary Table 1

Mass spectrometric identification of in-gel digested proteins from SDS-PAGE. Ten selected bands from the SDS-PAGE of Ap-3 fraction, labeled 1–10 in Fig. 1f were analyzed. The protein scores are calculated as the sum of the scores of peptides.

Protein component	Protein name	In-gel digestion of bands from fraction Ap-3 (score)									
		1	2	3	4	5	6	7	8	9	10
α-PC	CpcA							6,406	1,598		
β-PC	CpcB						4,738	755	520		
L_{HC}	CpcN	9,625	6,996								
L_{R}	CpcD									2,096	
L_{RC}	CpcJ			6,267							
L_{RC}	CpcG					3,266					
α-APC	ApcA1							223	1,843		
β-APC	ApcB1							275	1,351		
α-APC	ApcA2							685	2,699		
β-APC	ApcB2							402	3,101		
β-APC	ApcB3							1,363			
L_{C}	ApcC1										432
L_{C}	ApcC2										525
L_{CM}	ApcE		11,814								
L_{CC}	ApcH				5,888						

Supplementary Table 2

MS analysis of the *A. panamensis* PBS isolation fractions from sucrose gradients. The criteria for identifying a protein were two peptides and a false discovery rate below 0.01. The protein scores are calculated as the sum of the scores of peptides. Relative abundance is estimated protein abundance by spectral counting for PBS-related proteins. MW (kDa) = Molecular Weight assigned by the software used in the analysis, Mascot search engine 2.5.

Ap-3

Protein component	Protein name	Score	Coverage (%)	MW (kDa)	Relative abundance (%)
α-PC	CpcA	10,671	97.6	17.8	11.6
β-PC	CpcB	12,748	96.5	18.4	14.2
L_{HC}	CpcN	7,142	71.9	133.8	8.1
L_{R}	CpcD	5,603	100	8.7	5.7
L_{RC}	CpcJ	4,969	75.9	60.2	5.8
L_{RC}	CpcG	1,384	93.3	29	2.7
α-APC	ApcA1	2,994	99.4	17.3	3.9
β-APC	ApcB1	4,194	96.2	17.2	5.3
α-APC	ApcA2	7,320	99.4	17.4	11.8
β-APC	ApcB2	10,777	100	17.5	10.2
β-APC	ApcB3	1,035	89.4	17.6	1.4
L_{C}	ApcC1	1,274	95	6.8	1.6
L_{C}	ApcC2	912	92.7	7.8	1.6
L_{CM}	ApcE	8,019	82.7	127.7	10.9
L_{CC}	ApcH	10,777	94	48.7	4.9

Ap-2

Protein component	Protein name	Score	Coverage (%)	MW (kDa)	Relative abundance (%)
α-PC	CpcA	23,121	88.3	17.8	16.8
β-PC	CpcB	31,830	94.2	18.4	25.6
L_{HC}	CpcN	18,983	58.4	133.8	13.1
L_{R}	CpcD	14,790	84.6	8.7	7.2
L_{RC}	CpcJ	7,069	56.3	60.2	5.7
L_{RC}	CpcG	420	42.1	29	0.4
α-APC	ApcA1	4,840	80.1	17.3	4.1
β-APC	ApcB1	6,987	93.7	17.2	5.8
α-APC	ApcA2	7,024	90.7	17.4	6.7
β-APC	ApcB2	17,776	95.7	17.5	10.0
β-APC	ApcB3	434	46	17.6	0.6
L_{C}	ApcC1	304	73.3	6.8	0.3
L_{C}	ApcC2	126	42.7	7.8	0.2
L_{CM}	ApcE	1,893	33.6	127.7	1.6
L_{CC}	ApcH	2100	63.1	48.7	1.9

Ap-1

Protein component	Protein name	Score	Coverage (%)	MW (kDa)	Relative abundance (%)
α-PC	CpcA	15,005	57.7	17.8	22.6
β-PC	CpcB	17,841	78.5	18.4	32.8
L_{HC}	CpcN	1,115	18.3	133.8	1.5
L_R	CpcD	3,033	64.1	8.7	3.0
L_{RC}	CpcJ	1,697	29.2	60.2	2.7
L_{RC}	CpcG	306	26.2	29	1.2
α-APC	ApcA1	1,255	59	17.3	2.0
β-APC	ApcB1	615	50.6	17.2	1.5
α-APC	ApcA2	6,860	82	17.4	13.5
β-APC	ApcB2	15,143	61.7	17.5	17.1
β-APC	ApcB3	243	25.5	17.6	0.4
L_C	ApcC1	nd[a]	nd	6.8	nd
L_C	ApcC2	nd	nd	7.8	nd
L_{CM}	ApcE	678	14.5	127.7	1.4
L_{CC}	ApcH	247	13.7	48.7	0.4

nd[a]—not detected.

Supplementary Table 3

***A. panamensis* PBS subunits.** CpcN and ApcH, the two linkers that function as chain-core and core-core linkages, were first identified in this study.

Protein component	Protein name	NCBI accession number	Function	MW (kDa)
α-PC	CpcA	WP_218080329	PC α subunit	17.8
β-PC	CpcB	WP_218080328	PC β subunit	18.4
L_{HC}	CpcN	WP_245395491	Chain-core linker	133.8
L_R	CpcD	WP_218080330	Rod-capping linker	8.7
L_{RC}	CpcJ	WP_218080332	Rod-core linker	60.2
L_{RC}	CpcG	WP_218080233	Rod-core linker	29
α-APC	ApcA1	WP_218082489	APC α subunit	17.3
β-APC	ApcB1	WP_218082488	APC β subunit	17.2
α-APC	ApcA2	WP_218080831	APC α subunit	17.4
β-APC	ApcB2	WP_218080830	APC β subunit	17.5
β-APC	ApcB3	WP_218079828	APC β subunit	17.6
L_C	ApcC1	WP_218080829	Core linker	6.8
L_C	ApcC2	WP_218080828	Core linker	7.8
L_{CM}	ApcE	WP_218080833	Core-membrane linker	127.7
L_{CC}	ApcH	WP_218080832	Core-core linker	48.7

Supplementary Table 4
The bilin distances (Å) among the terminal emitters.

Strain	PBS shape[5]	α_{ApcE}-β_{ApcF} (Å)	α_{ApcE}-α_{ApcD} (Å)	Reference
Griffithsia pacifica	Block-shaped	20	29.9	19
Porphyridium purpureum	Hemiellipsoidal	19.3	31.8	18
Synechococcus sp. PCC 7002	Hemidiscoidal	19	29	8
Anabaena sp. PCC 7120	Hemidiscoidal	20	30	8
Syn6803	Hemidiscoidal	23	31	10
A. panamensis	Paddle-shaped	19.6[a]	32.1[b]	This study

a: $^{A'2}\alpha_{1\ ApcE}$-$^{A'2}\beta_{3\ ApcB2}$
b: $^{A'2}\alpha_{1\ ApcE}$-$^{A'1}\alpha_{1\ ApcA2}$

Supplementary Table 5
Summary of cryo-EM data collection and 3D reconstruction statistics

	Talos data set (Screen)	Titan data set (High resolution)
**Data collection**		
EM equipment	Talos Artica	Titan Krios
Voltage (kV)	200	300
Cs (mm)	2.7	2.7
Magnification (nominal)	92,000	81,000
Detector	Falcon 3EC	K3
Pixel size (Å)	1.0975	1.061
Electron exposure ($e^{-}/Å^{2}$)	~ 48	~ 50
Exposure time (s)	2.5	2.1
Frames (no.)	50	50
Defocus range (μm)	-0.5 ~ -3.1	-0.5 ~ -3.0
**Reconstruction**		
Software	cryoSPARC	cryoSPARC
Micrographs stacks (no.)	3,268	21,395
Final particle images (no.)	119,566	1,109,579
Symmetry imposed	C1 or C2	C1
Map final resolution (Å) *	5.56 (bin 2.5x)	2.99
Map sharpening B-factor (Å2)	N.D.	-103.8

* According to FSC=0.143

Supplementary Table 6
Cryo-EM data collection, refinement and validation statistics

	Cluster A† (EMD-35565) (PDB-8IMI)	Cluster B† (EMD-35566) (PDB-8IMJ)	Cluster C† (EMD-35567) (PDB-8IMK)	Cluster D† (EMD-35568) (PDB-8IML)
**Data collection and processing**				
Magnification	81,000x	81,000x	81,000x	81,000x
Voltage (kV)	300	300	300	300
Electron exposure (e–/Å^2)	~ 50	~ 50	~ 50	~ 50
Defocus range (μm)	-0.5 ~ -3.0	-0.5 ~ -3.0	-0.5 ~ -3.0	-0.5 ~ -3.0
Pixel size (Å)	1.061	1.061	1.061	1.061
Symmetry imposed	C1	C1	C1	C1
Initial particle images (no.)	1,966,948	1,966,948	1,966,948	1,966,948
Final particle images (no.)	1,109,579	1,109,579	1,109,579	1,109,579
Map resolution (Å)	2.59	2.59	2.48	2.74
FSC threshold	0.143	0.143	0.143	0.143
Map resolution range (Å)	2.34 ~ 10.0	2.32 ~ 10.0	2.34 ~ 10.0	2.32 ~ 10.0
**Refinement**				
Initial model used (PDB code)	Swissmodel & Alphafold2	Swissmodel & Alphafold2	Swissmodel & Alphafold2	Swissmodel & Alphafold2
Model resolution (Å)	2.5	2.5	2.3	2.9
FSC threshold	0.5	0.5	0.5	0.5
Model resolution range (Å)	1.5~2.5	1.5~2.5	1.4~2.3	1.7~2.9
Map sharpening *B* factor (Å^2)	88.88	89.13	83.13	98.67
Model composition				
Non-hydrogen atoms	69,972	69,972	68,848	55,864
Protein residues	8,859	8,859	8,750	6,968
Ligands	48	48	48	54
B factors (Å^2)				
Protein	35.87	37.79	32.57	44.71
Ligand	33.14	34.42	28.46	51.25
R.m.s. deviations				
Bond lengths (Å)	0.006	0.005	0.006	0.007
Bond angles (°)	1.123	1.118	0.868	1.102
Validation				
MolProbity score	1.59	1.60	1.48	1.72
Clashscore	11.87	12.32	9.06	14.41
Poor rotamers (%)	0	0	0	0
Ramachandran plot				
Favored (%)	98.25	98.16	98.84	97.76
Allowed (%)	1.75	1.84	1.16	2.24
Disallowed (%)	0	0	0	0

† **Cluster A:** A1-A2, A3-A4, B'1-B'2, C'1-C'2, linker: ApcE (1).
† **Cluster B:** A'1-A'2, A'3-A'4, B1-B2, C1-C2; linker: ApcE (2).
† **Cluster C:** D3-D4, D1-D2, D'3-D'4, D'1-D'2; linker: ApcH (1), ApcH (2).
† **Cluster D:** Rs2I-Rs2II, Rs1I-Rs1II, RbI-RbII; linker: CpcJ (1), CpcG (1).

Cryo-EM data collection, refinement and validation statistics

	Cluster E† (EMD-35569) (PDB-8IMM)	Cluster F† (EMD-35570) (PDB-8IMN)	Cluster G† (EMD-35571) (PDB-8IMO)
**Data collection and processing**			
Magnification	81,000x	81,000x	81,000x
Voltage (kV)	300	300	300
Electron exposure (e–/Å^2)	~ 50	~ 50	~ 50
Defocus range (μm)	-0.5 ~ -3.0	-0.5 ~ -3.0	-0.5 ~ -3.0
Pixel size (Å)	1.061	1.061	1.061
Symmetry imposed	C1	C1	C1
Initial particle images (no.)	1,966,948	1,966,948	1,966,948
Final particle images (no.)	1,109,579	1,109,579	1,109,579
Map resolution (Å)	2.76	3.07	3.08
FSC threshold	0.143	0.143	0.143
Map resolution range (Å)	2.42 ~ 10.0	2.29 ~ 10.0	2.73 ~ 10.0
**Refinement**			
Initial model used (PDB code)	Swissmodel & Alphafold2	Swissmodel & Alphafold2	Swissmodel & Alphafold2
Model resolution (Å)	2.9	3.4	3.3
FSC threshold	0.5	0.5	0.5
Model resolution range (Å)	1.7~2.9	2.0~3.4	2.0~3.3
Map sharpening *B* factor (Å^2)	96.03	133.20	139.30
Model composition			
Non-hydrogen atoms	55,813	55,489	55,489
Protein residues	6,961	6,924	6,924
Ligands	54	54	54
B factors (Å^2)			
Protein	56.38	112.23	92.91
Ligand	62.27	113.29	101.01
R.m.s. deviations			
Bond lengths (Å)	0.008	0.006	0.005
Bond angles (°)	1.156	1.096	0.890
Validation			
MolProbity score	1.81	2.00	2.09
Clashscore	15.01	20.78	23.32
Poor rotamers (%)	0	0	0
Ramachandran plot			
Favored (%)	97.32	96.86	96.52
Allowed (%)	2.68	3.14	3.48
Disallowed (%)	0	0	0

† **Cluster E:** Rs2'I-Rs2'II, Rs1'I-Rs1'II, Rb'I-Rb'II; linker: CpcJ (2), CpcG (2).
† **Cluster F:** Rt1I-Rt1II, Rt2'I-Rt2'II, Rt3I-Rt3II; linker: CpcN (1).
† **Cluster G:** Rt1'I-Rt1'II, Rt2I-Rt2II, Rt3'I-Rt3'II; linker: CpcN (2).

65 Watanabe, M., Sato, M., Kondo, K., Narikawa, R. & Ikeuchi, M. Phycobilisome model with novel skeleton-like structures in a glaucocystophyte *Cyanophora paradoxa*. *Biochim. Biophys. Acta - Bioenerg.* **1817**, 1428-1435 (2012).
66 Gan, F. *et al*. Extensive remodeling of a cyanobacterial photosynthetic apparatus in far-red light. *Science* **345**, 1312–1317 (2014).
