# Supplementary material for: A structure of the relict phycobilisome from a thylakoid-free cyanobacterium
Source: Nat Commun. 2023 Dec 4;14:8009. doi: 10.1038/s41467-023-43646-9 (PMC10696076; doi:10.1038/s41467-023-43646-9)
Supplement: Supplementary file 3 — Description of Additional Supplementary Files [file 41467_2023_43646_MOESM3_ESM.pdf]

Description of Additional Supplementary Files

File name: Supplementary Data 1
Description: List of the identified proteins of in-gel digested proteins from SDS-PAGE (Supplementary Table 1).

File name: Supplementary Data 2
Description: List of the identified proteins of PBS isolation fractions from sucrose gradients (Supplementary Table 2).
