# Supplementary material for: A structure of the relict phycobilisome from a thylakoid-free cyanobacterium
Source: Nat Commun. 2023 Dec 4;14:8009. doi: 10.1038/s41467-023-43646-9 (PMC10696076; doi:10.1038/s41467-023-43646-9)
Supplement: Supplementary file 6 — Reporting Summary [file 41467_2023_43646_MOESM6_ESM.pdf]

nature portfolio

Corresponding author(s): Ming-Yang Ho; Meng-Chiao Ho

Last updated by author(s): Oct 24, 2023

Reporting Summary

Nature Portfolio wishes to improve the reproducibility of the work that we publish. This form provides structure for consistency and transparency in reporting. For further information on Nature Portfolio policies, see our Editorial Policies and the Editorial Policy Checklist.

Statistics

For all statistical analyses, confirm that the following items are present in the figure legend, table legend, main text, or Methods section.

n/a	Confirmed	
☐	☒	The exact sample size (*n*) for each experimental group/condition, given as a discrete number and unit of measurement
☐	☒	A statement on whether measurements were taken from distinct samples or whether the same sample was measured repeatedly
☒	☐	The statistical test(s) used AND whether they are one- or two-sided<br>*Only common tests should be described solely by name; describe more complex techniques in the Methods section.*
☒	☐	A description of all covariates tested
☒	☐	A description of any assumptions or corrections, such as tests of normality and adjustment for multiple comparisons
☒	☐	A full description of the statistical parameters including central tendency (e.g. means) or other basic estimates (e.g. regression coefficient) AND variation (e.g. standard deviation) or associated estimates of uncertainty (e.g. confidence intervals)
☒	☐	For null hypothesis testing, the test statistic (e.g. *F*, *t*, *r*) with confidence intervals, effect sizes, degrees of freedom and *P* value noted<br>*Give P values as exact values whenever suitable.*
☒	☐	For Bayesian analysis, information on the choice of priors and Markov chain Monte Carlo settings
☒	☐	For hierarchical and complex designs, identification of the appropriate level for tests and full reporting of outcomes
☒	☐	Estimates of effect sizes (e.g. Cohen's *d*, Pearson's *r*), indicating how they were calculated

Our web collection on statistics for biologists contains articles on many of the points above.

Software and code

Policy information about availability of computer code

Data collection	Absorption spectrum were collected using Cary 60 WinUV (5.1.3.1042). Fluorescence emission spectra were collected using FL Solutions for F-7000. Cryo-EM data were acquired on a Titan Krios G3 (Thermo Fisher) microscope operated at 300 KeV, equipped with a GIF Quantum K3 dector system (Gatan) or a Talos Arctica (Thermo Fisher) microscope operated at 200 KeV, equipped with a Falcon III detector. Automated data acquisition was carried out using EPU-2.7.0 (Thermo Fisher).
Data analysis	MotionCor2-1.4.4, CTFFind4-4.1.14, UCSF Chimera-1.15, Alphafold2-v1.5.2, WinCOOT-0.8.6.1, PHENIX-1.18.2-3874, cryoSPARC-3.3.2., R-4.2.1, IQ-TREE 2.1.2, MEGA X, Origin 2020b (9.7.5.184), Mascot search engine 2.5, Gaussian 09, GaussView 5.0.9, Multiwfn-3.8dev

For manuscripts utilizing custom algorithms or software that are central to the research but not yet described in published literature, software must be made available to editors and reviewers. We strongly encourage code deposition in a community repository (e.g. GitHub). See the Nature Portfolio guidelines for submitting code & software for further information.

Data

Policy information about availability of data

All manuscripts must include a data availability statement. This statement should provide the following information, where applicable:

- Accession codes, unique identifiers, or web links for publicly available datasets
- A description of any restrictions on data availability
- For clinical datasets or third party data, please ensure that the statement adheres to our policy

The cryo-EM map of PBS megacomplex structure been deposited in the Electron Microscopy Data Bank (EMDB) with accession codes: EMD-35530.
Twenty-four focus-refined PBS cylinder cryo-EM maps have been deposited in the EMDB with accession codes: EMD-35531, EMD-35534, EMD-35537, EMD-35541, EMD-35542, EMD-35544, EMD-35546, EMD-35548, EMD-35549, EMD-35550, EMD-35551, EMD-35552, EMD-35553, EMD-35554, EMD-35555, EMD-35556, EMD-35557, EMD-35558, EMD-35559, EMD-35560, EMD-35561, EMD-35562, EMD-35563 and EMD-35564.
Seven artificially stitched maps have been deposited in the EMDB with the accession codes: EMD-35565, EMD-35566, EMD-35567, EMD-35568, EMD-35569, EMD-35570, EMD-35571.
Seven atomic coordinates have been deposited in the PDB with the accession codes: 8IMI, 8IMJ, 8IMK, 8IML, 8IMM, 8IMN, 8IMO.

Research involving human participants, their data, or biological material

Policy information about studies with human participants or human data. See also policy information about sex, gender (identity/presentation), and sexual orientation and race, ethnicity and racism.

Reporting on sex and gender	This is not applicable to because our study did not include human.
Reporting on race, ethnicity, or other socially relevant groupings	This is not applicable to because our study did not include human.
Population characteristics	This is not applicable to because our study did not include human.
Recruitment	This is not applicable to because our study did not include human.
Ethics oversight	This is not applicable to because our study did not include human.

Note that full information on the approval of the study protocol must also be provided in the manuscript.

Field-specific reporting

Please select the one below that is the best fit for your research. If you are not sure, read the appropriate sections before making your selection.

☒ Life sciences ☐ Behavioural & social sciences ☐ Ecological, evolutionary & environmental sciences

For a reference copy of the document with all sections, see nature.com/documents/nr-reporting-summary-flat.pdf

Life sciences study design

All studies must disclose on these points even when the disclosure is negative.

Sample size	We have conducted multiple rounds of purification and characterization of PBS using sucrose density gradient centrifugation, SDS-PAGE, absorption spectrum, and fluorescence emission spectra.No sample-size calculations were performed. Sample size was chosen based on previous experience and standards in the field. All experiments were repeated at least three times and produced consistent results. Additionally, we analyzed two batches of samples using LC-MS/MS, which confirmed the consistency of our purification method. Regarding cryo-EM data, we collected sufficient sample sizes, including the number of images and extracted particles, until we could obtain high-resolution reconstructed maps, estimated by Fourier shell correlation. The amount of cryo-EM micrographs collected was determined based on our prior knowledge that the reconstruction of the protein particles picked from these micrographs could reach near-atomic resolution.
Data exclusions	The cryo-EM analysis involved exclusion of particles that did not improve the map quality, using standard classification procedures in cryoSPARC. Only data that passed quality control was included in the analysis. 2D and 3D classification were performed to identify classes with clear structural signals and intact structures, and only particles in these classes were selected for the final reconstruction and refinement. During raw micrograph screening, images were excluded based on quality. Particle selection criteria were based on the quality of resulting 2D class average and 3D maps.
Replication	The spectral measurements were performed in three replicates, giving the similar results.
Randomization	This is not relevant to our study because we did not treat our samples in various conditions.

Blinding	Blinding is not essential to a cryo-EM experiment and biochemical assay, because we are studying a specific protein complex.

Reporting for specific materials, systems and methods

We require information from authors about some types of materials, experimental systems and methods used in many studies. Here, indicate whether each material, system or method listed is relevant to your study. If you are not sure if a list item applies to your research, read the appropriate section before selecting a response.

Materials & experimental systems

n/a	Involved in the study
☒	☐ Antibodies
☒	☐ Eukaryotic cell lines
☒	☐ Palaeontology and archaeology
☒	☐ Animals and other organisms
☒	☐ Clinical data
☒	☐ Dual use research of concern
☒	☐ Plants

Methods

n/a	Involved in the study
☒	☐ ChIP-seq
☒	☐ Flow cytometry
☒	☐ MRI-based neuroimaging
